# Chemoautotrophic production of gaseous hydrocarbons, bioplastics and osmolytes by a novel *Halomonas* species

**DOI:** 10.1186/s13068-023-02404-1

**Published:** 2023-10-11

**Authors:** Matthew Faulkner, Robin Hoeven, Paul P. Kelly, Yaqi Sun, Helen Park, Lu-Ning Liu, Helen S. Toogood, Nigel S. Scrutton

**Affiliations:** 1 Manchester Institute of Biotechnology, The University of Manchester, 131 Princess Street, Manchester, M1 7DN UK; 2 Institute of Systems, Molecular and Integrative Biology, University of Liverpool, Biosciences Building, Crown Street, Liverpool, L69 7BE UK; 3 C3 Biotechnologies Ltd, 20 Mannin Way, Caton Road, Lancaster, LA1 35W Lancashire UK; 4 Present Address: Engineering Building A, University of Manchester, Oxford Road, Manchester, M13 9PL UK

## Abstract

**Background:**

Production of relatively low value, bulk commodity chemicals and fuels by microbial species requires a step-change in approach to decrease the capital and operational costs associated with scaled fermentation. The utilisation of the robust and halophilic industrial host organisms of the genus *Halomonas* could dramatically decrease biomanufacturing costs owing to their ability to grow in seawater, using waste biogenic feedstocks, under non-sterile conditions.

**Results:**

We describe the isolation of *Halomonas rowanensis*, a novel facultative chemoautotrophic species of *Halomonas* from a natural brine spring. We investigated the ability of this species to produce ectoine, a compound of considerable industrial interest, under heterotrophic conditions. Fixation of radiolabelled NaH^14^CO_3_ by *H. rowanensis* was confirmed in mineral medium supplied with thiosulfate as an energy source. Genome sequencing suggested carbon fixation proceeds via a reductive tricarboxylic acid cycle, and not the Calvin–Bensen–Bassham cycle. The mechanism of energy generation to support chemoautotrophy is unknown owing to the absence of an annotated SOX-based thiosulfate-mediated energy conversion system. We investigated further the biotechnological potential of the isolated *H. rowanensis* by demonstrating production of the gaseous hydrocarbon (bio-propane), bioplastics (poly-3-hydroxybutyrate) and osmolytes (ectoine) under heterotrophic and autotrophic CO_2_ fixation growth conditions.

**Conclusions:**

This proof-of-concept study illustrates the value of recruiting environmental isolates as industrial hosts for chemicals biomanufacturing, where CO_2_ utilisation could replace, or augment, the use of biogenic feedstocks in non-sterile, industrialised bioreactors.

**Supplementary Information:**

The online version contains supplementary material available at 10.1186/s13068-023-02404-1.

## Highlights

- New halophilic isolate *Halomonas rowanensis* is useful for chemicals and fuel production at neutral pH.
- *H. rowanensis* is a likely facultative chemolithotroph capable of CO_2_ fixation in the presence of thiosulfate.
- Propane production occurred under both heterotrophic and autotrophic growth conditions with recombinant *H. rowanensis*.
- Potential application of *H. rowanensis* in the non-sterile production of chemicals and advanced synthetic fuels.

**Supplementary Information:**

The online version contains supplementary material available at 10.1186/s13068-023-02404-1.

## Background

Implementation of a bio-based economy relies on the development of efficient microbial bioprocesses to produce commodity chemicals and fuels. Advances in metabolic pathway and microbial host engineering have led to an ever-expanding toolbox that is enabling the development and optimisation of biological production routes to a diverse array of compounds. These include oleochemicals [1], gaseous fuels [2], petrochemical derivatives [3], biomaterials [4] and bioplastics [5]. Bio-production, however, is hampered by the relatively high cost of microbial fermentations. This is a major barrier to commercialisation, which prevents cost competitiveness with incumbent players in the petrochemical industries [6]. Microbial fermentations often require expensive raw materials, large quantities of fresh water, high energy usage, and challenging upstream and downstream process engineering [7]. A step-change in fermentation technology advancement is needed to reduce the capital and operational costs of biomanufacturing. If realised, this will overcome economic barriers, unlocking major societal and environmental benefits, and enable decentralised/distributed bioproduction industries to emerge from, and ultimately displace, the global dominance of fossil-based manufacturing industries.

The development of cost effective fermentation processes may require a non-traditional microbial chassis, such as organisms that can grow under extreme conditions (extremophiles) [8–10]. One example is the robust halophilic and alkaliphilic industrial host *Halomonas bluephagenesis* [11]. This bacterium is capable of growth in seawater or waste water of high salinity and alkalinity (pH 9–11) under non-sterile conditions, without microbial contamination [7, 12]. Under these conditions, *H. bluephagenesis* has been shown to grow continuously for polyhydroxyalkanoate (PHA) production at an industrial scale (> 1000 tonnes) for over 3 years without any decline in its growth potential [12]. This led to an approximate 65% cost saving of the overall pilot scale bioprocess compared with using *Escherichia coli* as the production host [13]. In addition, bioproduction plants are not dependent on the use of expensive, steel-based bioreactors, sanitising systems, or a requirement for freshwater [11, 14]. Genetic toolboxes for halophilic organisms already exist [2, 15–18], which have enabled the production of a range of products, such as ectoine, by *H. bluephagenesis* under non-sterile conditions [2, 16, 19].

Production of bio-LPG (propane, butane and isobutane) by *H. bluephagenesis* has been reported at laboratory scale [2, 20] and initial technoeconomic analysis was performed using locally sourced seawater and a low cost industrial waste feedstock (glycerol derived from biodiesel manufacture; [2]). However, this strain was not an ideal host, as propane production only occurred at near neutral pH, near the limits of the pH range capable of supporting growth [2, 20]. An alternative low organic carbon-based approach to gaseous hydrocarbon production was also demonstrated, using an engineered photosynthetic microorganism *Synechocystis* sp. PCC6803 to generate propane direct from CO_2_, albeit at low titre and productivity (Amer et al. [20]). These findings motivated us to search for *Halomonas* strains capable of growth at neutral pH, ideally using chemoautotrophic CO_2_ fixation to support chemicals production. This is not without precedent as chemoautotrophic growth of *Halomonas stevensii* has been demonstrated using thiosulphate as the energy source [21]. We selected the ‘Old Biot’ neutral pH brine spring in Nantwich (Cheshire, UK) as the source of environmental samples to screen for facultative chemoautotrophs to enable us to isolate halophiles capable of cultivation on more than one waste carbon source. These strains served as new potential microbial chassis to produce bio-propane, ectoine and PHA under heterotrophic and/or chemoautotrophic conditions.

## Materials and methods

### Materials, plasmids and strains

All chemicals and solvents were commercially sourced and were of analytical grade or better. Media components were purchased from Formedium (Norfolk, UK). Gene sequencing and oligonucleotide synthesis were performed by Eurofins MWG (Ebersberg, Germany) and Integrated DNA Technologies (Iowa, USA). Plasmids for propane production used in this study were pHAL7–CvFAP_G462V_ and pHAL102–CvFAP_G462V_ (Additional file 1: Fig. S1) [17]. These vectors contain the propane producing gene encoding fatty acid photodecarboxylase (CvFAP) from *Chlorella variabilis* NC64A variant G462V [2]. They also contain a broad-host-range origin of replication (oriV) from *Pseudomonas aeruginosa*, as well as an origin of transfer (oriT) and either a weak or strong constitutive promoter, respectively [17].

The *E. coli* strains S17-1 [22] and BL21(DE3) (New England Biolabs) were used for plasmid conjugation into *Halomonas* and radiolabelled CO_2_ fixation assays, respectively. *Salmonella enterica* and *Halothiobacillus neapolitanus* used for the ^14^C fixation study were kindly supplied by Prof Liu (University of Liverpool, UK). Native *H. bluephagenesis* TD01 was previously isolated from the Aydingkol Lake in Xinjian, China [23]. The engineered *H. bluephagenesis* strain TD1.0 is a derivative with a genomic encoded T7-like MmP1 system for IPTG inducible recombinant protein expression *H. bluephagenesis* strain TQ10 lacking genes required for PHB production is a subsequent TD1.0 derivative [2, 18, 20]. These strains were kindly supplied by Professor Guo-Qiang Chen (Tsinghua University, China).

Environmental water samples originating from the Old Biot brine spring in Nantwich (Cheshire, UK) were collected into sterile bottles and stored at 4 °C. Brine (1 L) was filtered (0.2 µm) to remove debris and microorganisms. Multiple debris-free regions of the filter paper were sampled for microorganism content by touching with a sterile inoculation loop and resuspending in 10 mL sterilized brine filtrate. Serial dilutions (10-, 100- and 1000-fold) of the samples in sterile brine filtrate were performed, and aliquots were cultivated on LB agar plates containing 5% (w/v) NaCl at 30 ºC for several days until colonies appeared in the culture plates. Individual colonies were repeatedly picked and re-streaked on fresh plates to ensure isolate homogeneity. Glycerol stocks were generated of each isolate by combining equal volumes of overnight cultures with 50% sterile glycerol and flash freezing in liquid nitrogen.

### Growth medium and standard cultivation

Standard cultivation of *E. coli* and *S. enterica* subsp. enterica serovar Hillingdon ATCC9184 were performed in Luria broth (LB; 5 g/L yeast extract, 10 g/L tryptone and 10 g/L NaCl pH 7.0) at 37 °C for 16–24 h. High salt Luria broth (LB60; LB containing 60 g/L NaCl) was used routinely for heterotrophic cultivation of *Halomonas* strains at pH 6.8 (propane production) or 9.0 (conjugation and recombinant enzyme expression) at 30 °C. Strain characterization studies were performed primarily in LB60 medium, varying the concentration of NaCl [1–20% (w/v)], pH (6–10) and supplemental butyric acid (0–80 mM). Growth of *Halomonas* strains was additionally performed in environmental water samples with or without supplemental glycerol [0.5% (w/v)]. Environmental water was obtained from three sources within the Greater Manchester region (Medlock and Mersey rivers and the Rochdale canal; UK). This was subsequently filter sterilised and supplemented with NaCl to obtain a final 6% (w/v) concentration (by refractometer). An additional high salinity minimal medium was utilised for *Halomonas* cultivation consisting of filter sterilised sea water (Irish sea) with or without a supplemental carbon source (0.5% (w/v) glycerol).

Chemoautotrophic growth of new isolates and control strains was performed in thiosulfate minimal medium pH 7.0 (1 g/L KNO_3_, 1 g/L KH_2_PO_4_, 0.5 g/L NH_4_Cl, 15 g/L Na_2_S_2_O_3_ and 60 g/L NaCl pH 7.0) at 30 °C for 48 h with 180 rpm agitation. The initial cultivation of *H. neapolitanus* was performed in ATCC 290 medium for thiobacilli (1.2 g/L Na_2_HPO_4_, 1.8 g/L KH_2_PO_4_, 0.1 g/L MgSO_4_.7H_2_O, 0.1 g/L (NH_4_)_2_SO_4_, 0.03 g/L CaCl_2_, 0.02 g/L FeCl_3_ and 0.02 g/L MnSO_4_) supplemented with sodium thiosulfate (10 g/L) at 30 °C for 24 h with 180 rpm agitation.

### New isolate identification and genome sequencing

Classification of each Old Biot brine spring isolates was performed by 16S rDNA analysis of individual colonies. PCR was performed using primers specific for the 16S genomic hypervariable region, (*aggagatataccatg*CCTACGGGNGGCWGCAG) and (*tggtggtgctcgag-*GACTACGGGTATCTAATCC), which contained 5’ overhangs (lower case italics) to allow PCR products to be cloned into the pET-28b(+) vector. The latter was performed using In-Fusion cloning (Takara Bio) with pET-28b linearised by PCR (primers: CTCGAGCACCACCACCACC and CTCTAGAAATAATTTTGTTTAACTTTAAGAAGG-AGATATACCatg). Partial 16S rDNA sequences were determined by plasmid sequencing, and species identity was inferred by homology using the EMBL phylogeny tools (https://www.ebi.ac.uk/) [24]. One fast growing isolate (I5) was identified as a *Halomonas* species and named *Halomonas rowanensis*.

A partial genome sequencing of isolate *H. rowanensis* was performed by MicrobesNG (Birmingham, UK) using DNA isolated from cultures grown on LB60 agar plates. Subsequent high coverage full genome sequence data for this strain was obtained by in house PacBio Sequel next generation sequencing using DNA purified from overnight cultures in LB60 using a Monarch genomic DNA isolation kit (New England Biolabs). In this process, genomic DNA was sheared to generate approximately 10 kb fragments using g-TUBES (Covaris) following the manufacturer’s instructions. DNA quality was verified using a Fragment Analyzer using the DNF-930 protocol (Agilent). The Express Template Prep Kit 2.0 procedure was used to process the samples for sequencing with multiplexing using the barcoded overhang adapter kit (Pacific Biosciences). Data were acquired using the Pacific Biosciences Sequel system. Following demultiplexing the genomic sequences were assembled using the Resequencing algorithm of SMRT Link 8.0. This yielded mean coverage depth of 280 of two assembled contigs 3,812,367,3 and 229,491 bp, with around 64% of the annotated proteins identified and ~ 36% hypothetical annotated files were inspected for quality of annotation and the presence of known biosynthetic pathways using Artemis [25] and Patrik [26]. A list of the annotated genes from *H. rowanensis* is found in the Additional file 1: Data file. The latest annotated *H. rowanensis* genome sequence is available on Genbank (Taxonomy ID: 3,061,632, Bioproject ID PRJNA994417) and ENA (Taxonomy ID: 3,049,753, Study ID: PRJEB64241, Study Title: ERP149380), project number PRJNA994417) as is *Halomonas bluephagensis* TD01 (Genbank, GCA_923868895.1 and GCA_000219565.1).

### Phylogenetic analysis

16S rDNA sequences of the isolates were aligned using MUSCLE (v. 3.7) and trimmed using TrimAl (v. 1.3). The data were used to generate a distance matrix with DnaDist (Phylip v. 3.68) [27], via the Phylemon2 web portal (http://phylemon.bioinfo.cipf.es/index.html) [28]. Phylogenetic trees in Newick format were generated using the Fitch-Margoliash method via WebPhylip [29] and trees were visualised with ETE Toolkit TreeView [30].

### Metabolic pathway mapping

Annotated PacBio genome sequences were used to build a new database within the PathoLogic tool of the BioCyc Pathway Tools (v 23.0; P10) [31]. Default settings were used during the database initialisation, replicon specification and build to identify metabolic pathways from the genome data. These pathways were then used to inform manual homology searches and improve genome annotation through NCBI BLASTp (https://blast.ncbi.nlm.nih.gov/Blast.cgi) with varied parameters decided based on the similarity of the sequence in question with those in the database.

### Structure prediction and structure homology searches

Validation of the annotation of putative carbon fixation and thiosulfate utilisation pathway genes in the genome sequence of *H. rowanensis* (Additional file 1: Fig. S2) was performed by generating three dimensional models using the Alphafold 2.0 server [32]. This was performed using the recommended default parameters for prokaryotic peptide sequences. These structures were viewed and interrogated in Pymol [33]. The DALI webserver [34] was used to match the predicted structures to the entire PDB database by homology with the recommended default settings. The resulting match with the lowest RMSD alignment was viewed and manual alignments performed in Pymol. The resulting alignment was interrogated for comparison of key residues from the predictions in the active site or those interacting with bound ligands in the published PDB structures.

### Quantitation of ectoine and PHA production

Ectoine was harvested from *Halomonas* cultures using a modified ‘bacterial milking’ method described previously [35]. Cultures (50 mL) were harvested by centrifugation at 4000*g* for 5 min at room temperature. The pellet was resuspended in 5 mL of ultrapure water (18.2 MΩ·cm) and vortexed for 1 min until homogeneous. The suspension was incubated for 10 min at room temperature and centrifuged as before. The milky white supernatant was retained and the ectoine content was quantified by HPLC using an Agilent 1260 Infinity HPLC with a 1260 ALS autosampler, TCC SL column heater, a 1260 refractive index detector (RID). Samples were run on an Agilent Hi-Plex-H column (300 × 7.7 mm) using 2.5 mM sulfuric acid as the mobile phase (0.6 mL/min) at 60 °C for 30 min. Ectoine quantitation (retention time of 6.3 min) was performed by comparing peak areas to a standard curve generated from authentic standards. A salinity gradient in the thiosulfate medium was used to find the optimum for ectoine production which is salinity dependent. Different water sources with a variety of minerals were tested as well as salinity from added NaCl.

PHA production of *H*. *rowanensis* and *H*. *bluephagenesis* TD1.0 were performed using a modification of the method described previously [23]. Cultures (50 mL) in growth medium (LB60 ± 4% (w/v) glucose and/or thiosulfate medium) were incubated at 37 °C with 180 rpm agitation for 48 h. Aliquots (50 mL) were centrifuged at 4000*g* for 10 min, followed by two cell pellet washes in 5 mL water with centrifuging as before. The pellets were flash frozen in liquid nitrogen and freeze dried overnight. Dried cell pellet (12–86 mg without glucose, 85–190 mg with glucose feed) in 4 mL airtight glass vials were dissolved in 1 mL chloroform, followed by the addition of the derivatization reagent (1 mL; 3% H_2_SO_4_ in anhydrous methanol containing the internal standard 0.1% benzoic acid). Mixtures were incubated at 100 °C in a sand bath for 4–6 h then cooled to room temperature. Sodium bicarbonate (~ 1 g) was added to quench the reaction and the samples were refrigerated overnight to allow any cell debris to precipitate. The debris was centrifuged at 4000 g and the supernatant was clarified by passage through a filter tip. The methyl 3-hydroxybutyrate content (derivatized PHA monomer) was determined by GC using an Agilent Technologies 7890A GC equipped with an FID detector using an HP-5 column [36]. Compound identification was determined by comparison of the retention time with authenticated standards run under identical conditions. PHA content is expressed as the percentage of PHA mass as a fraction of the total dry cell weight (DCW) of the lyophilized cell pellet (% g/g DCW).

### Radiometric bicarbonate uptake and fixation

*Halomonas* species were grown in LB60 or the thiosulfate medium [21], while ATCC290-medium for thiobacilli was used for growth of *H. neapolitanus*. Both *E. coli and S. enterica* were grown in LB medium. Cell cultures were grown to exponential phase, harvested by centrifugation, and were resuspended in Rubisco assay buffer (100 mM EPPS, pH 8.0, and 20 mM MgCl_2_). The ^14^C (H^14^CO_3_^−^) fixation assay was performed as described previously [37].

Cell cultures were grown to exponential phase using the medium above, harvested by centrifugation and resuspended in filtered nitrogen-bubbled culture medium to OD 600 nm of 4. The whole cell ^14^C uptake assay was performed according to previous studies [37] with several modifications. Radiolabelled NaH^14^CO_3_ (10 mM final concentration) was added, and the cultures were incubated at 37 °C for 10, 15 and 60 min. Cell permeabilization was performed by the addition of alkyltrimethylammonium bromide (MTA) to a final concentration of 0.03% (w/v). Assay termination was performed by adding formic acid [10% (v/v)] and boiling the mixtures on heating blocks (98 °C) until dry, removing any unconverted NaHCO_3_ as CO_2_ gas. The powders were resuspended in water (100 µl), mixed with scintillation cocktails (1 mL; Ultima Gold XR, PerkinElmer) and counted for radioactivity (Tri-Carb, PerkinElmer). Three biological repeats were performed for each sample and the data were normalised to the cell density (OD_600_ nm).

### Propane production by *H. rowanensis* and *H. bluephagenesis* TD1.0

Introduction of plasmids pHAL7–CvFAP_G462V_ and pHAL102–CvFAP_G462V_ [17] into *H. rowanensis* was performed via conjugation, using the S17-1 *E. coli* donor strain, as described previously [2]. Propane assays were performed as described previously for *Halomonas* cultures, except the medium (20 mL) was either LB60 or thiosulfate medium at pH 7.0 and the butyric acid concentration was 10 mM [2]. Propane quantitation was performed by headspace sampling using an Agilent 490 Micro GC [2]. *Halomonas* TD1.0 propane assays were as previously described in LB60 or thiosulfate medium at pH 9 [2].

## Results and discussion

### Isolates identification and genome sequencing

**Fig. 1:**
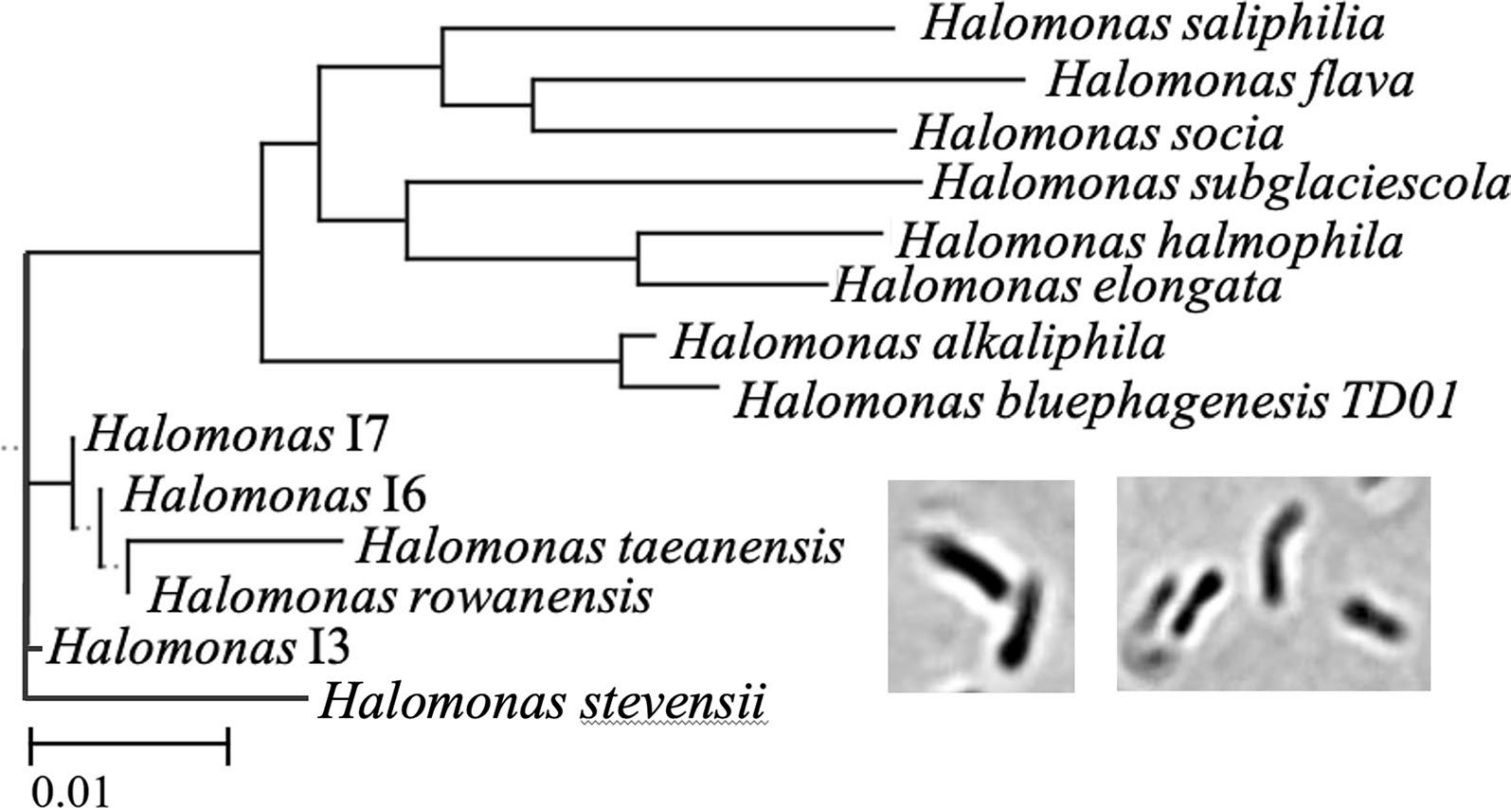
Phylogenetic tree of ‘Old Biot’ *Halomonas* isolates. Analysis using ^a^partial and ^b^full 16S rDNA sequences from PCR products and genome sequencing, respectively. Insets: light microscope images of *H. rowanensis* at 100X magnification. Strains: *H. saliphila* st. LCB169; *H. flava* st. YIM 94343; *H. socia* st. NY-011 16S; *H. subglaciescola* st. DSM 4683; *H. halmophila* st. ATCC 19717; *H. elongata* st. 1H9; *H. alkaliphilia* st. 18bAG and *H. taeanensis* st. BH539

A variety of native halophilic or halotolerant bacteria were isolated from a natural brine spring (pH 7.1) by enriching with heterotrophic culture medium specific for *H. bluephagenesis* [23], but at neutral pH. Partial 16S rDNA, sequence analysis of individual isolates revealed the presence of some Gram-positive *Bacillus* species (orange-coloured colonies) and a *Kocuria sp*. strain (small, white colonies). In addition, Gram-negative bacteria were present, including two potential *Idomarina* species (I9–I10) and several *Halomonas* species (isolates I3–I7) (Additional file 1: Table S1). The *Halomonas* isolates were small aerobic non motile rods (Fig. 1 inset), that grew heterotrophically on amino acid-based carbon sources.

Partial genome sequences were obtained for *Halomonas* I4 and *Idiomarina* I9 isolates, with a more complete genome sequence generated for the fastest growing *Halomonas* strain I5 (66.5% annotated). This compares to the 89% annotation of the *H. bluephagenesis* TD01 strain (Genbank: GCA_923868895.1), although in the latter case a secondary correlation of the low sequence identity genes needs to be performed. The 16S rRNA genes for *Halomonas* strains I4 and I5 were identical, with a 99% identity to the known organism *Halomonas taeanensis* strain BH539 [38] (Fig. 1). This strain was more distinct from the robust industrial strain *H. bluephagenesis* TD1.0, which prefers a highly alkaline environment. We designated the I5 (and by homology I4) strain as *Halomonas rowanensis*.

For the two *Idiomarina* isolates, the 16S rDNA sequences differed in 2 base positions, suggesting two different species or strains were present. We decided to take forward the *Halomonas* isolates as chassis for recombinant propane production as the synthetic biology toolbox is more advanced than for *Idiomarina*, and our existing synthetic biology constructs were already tailored for *Halomonas* [2, 17, 20].

### High salt tolerance of the new isolates

Each *Halomonas* isolate was screened for its tolerance towards salinity (NaCl) and optimal pH. The isolates were found to tolerate higher NaCl concentrations than the control *H. bluephagenesis* TD1.0 strain (Additional file 1: Fig. S2) but preferred neutral pH at higher salinities (9–12% NaCl). This is consistent with the naturally high salinity (~ 20%) and neutral pH observed for the native spring they were isolated from. Given our ultimate aim of biological propane production, we additionally tested the *Halomonas* isolates for tolerance towards the precursor butyric acid [2]. The highest butyric acid tolerance was found with *H. rowanensis* (80 mM), similar to engineered *H. bluephagenesis* TQ10 [20] and TD1.0 [36] (Additional file 1: Fig. S2).

The mechanism(s) of salt tolerance of the new isolates was investigated by genome mining for pathways known to be involved in osmoregulation. Genes associated with salt tolerance in *Halomonas biemenensis* include the sodium-translocating NADH:quinone oxidoreductase (nqrA) and an NAD-specific glutamate dehydrogenase (gdhB). These genes are associated with sodium efflux and the production of the secondary compatible solute glutamate [15]. Both genes were found to be present in the genomes of both the *Halomonas* and *Idiomarina* isolates I4, I5, I9 and I10.

### Production of compatible solutes and PHA

**Fig. 2:**
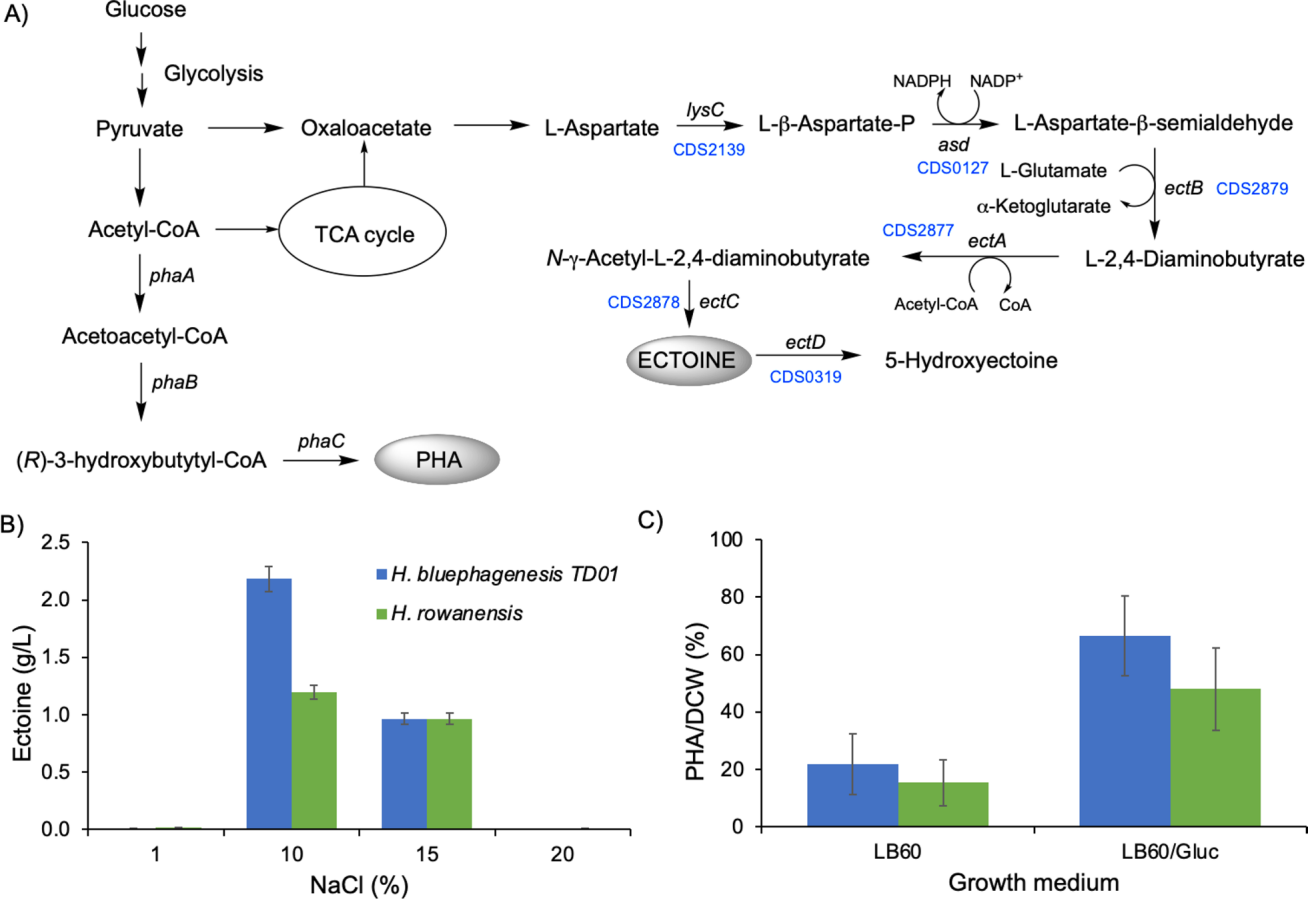
Heterotrophic ectoine and PHB production of *H. rowanensis*. **A** *Halomonas* metabolic pathway to ectoine and PHB production. Gene annotations from *H. rowanensis* are shown in blue. **B** Ectoine production of *H. rowanensis* (green) and *H. bluephagenesis* TD1.0 (blue) in variable levels of salinity. Cultures were harvested and milked for ectoine with ultrapure water as described previously [35]. **C** PHB production of *H. rowanensis* and *H. bluephagenesis* TD1.0 in LB60 with and without 4% glucose. Cultures were grown for 24 h and the PHA concentration was determined by polymer extraction, degradation, derivatization and GC quantitation. Error bars represent one standard deviation of triplicate analyses. Enzymes: asd = aspartate semialdehyde dehydrogenase 1; EctA = L-2,4-diaminobutyric acid acetyltransferase; ectB = L-2,4-diaminobutyric acid transaminase; ectC = L-ectoine synthase; ectD = ectoine dioxygenase; lysC = aspartate kinase; phaA = 3-ketothiolase; phaB = NADPH-dependent acetoacetyl-CoA reductase; phaC = PHA synthase. The PHB titre and CDW data are available in Additional file 1: Table S2

*Halomonas* species are known to counteract osmotic pressure by the production and intracellular accumulation of significant levels of the compatible solute ectoine [39]. This compound is a valuable product within biotechnology and cosmetics industries and has many medicinal uses. For example, ectoine is used as biofunctional stabilizers, skin protectors and potential drugs for diseases, such as Alzheimer’s and rhinoconjunctivitis [40]. Annotation of the *H. rowanensis* genome revealed the presence of a likely ectoine biosynthesis pathway (Fig. 2a). This included the *ectABC* operon and genes for aspartate kinase (*lysC*) and aspartate semialdehyde dehydrogenase 1 (*asd1*) [40]. In addition, the gene for ectoine dioxygenase (*ectD*) was present, which produces hydroxyectoine from ectoine. The osmoregulated solute TRAP transporter (*teaABC*) was also identified, which mediates the uptake of ectoine and hydroxyectoine in *Halomonas elongata* [41].

Ectoine can be obtained naturally from *H. elongata* by a ‘bacterial milking’ process [35]. We performed bacterial milking on *H. rowanensis* and a control *H. bluephagenesis* TD1.0 strain, the latter of which is known to generate large quantities of ectoine [12]. As expected, high quantities of ectoine were produced by both *Halomonas* strains under high salt conditions (10–15% NaCl; Fig. 2b). Both strains generated similar titres of ectoine at 15% salinity (0.97 g/L), comparable to titres achieved by *H. elongata* [35]. However, *H. bluephagenesis* TD1.0 generated nearly double the ectoine levels than *H. rowanensis* at only 10% NaCl (2.19 vs 1.20 g/L, respectively).

*Halomonas* species are also well-known as prolific PHA producers, generating up to 92% of dry cell weight (DCW) in strains engineered to upregulate the expression of the three native PHA genes (*phaA*, *phaB* and *phaC*; Fig. 2a [18]). The native PHA production titres of *H. rowanensis* in standard growth medium were marginally lower than *H. bluephagenesis* TD1.0 (15.38 ± 8.01% vs 21.90 ± 10.56%, respectively; Fig. 2c), with the low titres representative of harvesting long after the carbon source was depleted. Significantly higher titres were obtained when cultivated in the presence of high glucose levels, with *H. rowanensis* accumulating PHA at 48.05% ± 14.37% of the dry cell weight (Fig. 2c). This is equivalent to around 3.0 g/L PHA. Under the same growth conditions, *H. bluephagenesis* TD1.0 generated PHA at around 3.3 g/L. Therefore, *H. rowanensis* has a similar capacity as a PHA producer as the known industrial chassis *H. bluephagenesis* TD1.0, which is known to generate up to 82% DCW of PHA without any pathway engineering of the PHA biosynthesis operon [16, 23] and with multiple coproducts [36].

### Chemoautotrophic growth of *H. rowanensis* from CO_2_ in wastewater

A key advantage of using *Halomonas* as an industrial microbial chassis is its ability to grow under non-sterile conditions in sea water and wastewater using simple inexpensive organic carbon sources [2]. We extended this approach further by testing *H. rowanensis* for chemoautotrophic growth on salinity adjusted seawater (Irish sea) and polluted river or canal water collected from waterways around the Greater Manchester region [42]. Significant growth was detected with *H. rowanensis* after repeated re-streaking into sterile domestic waterway samples, but not seawater, in the absence of exogenous carbon sources. This suggested either significant organic carbon sources were naturally present in the Manchester waterways or *H. rowanensis* is capable of chemoautotrophic growth on inorganic carbon (e.g., CO_2_). Control cultivations of *H. bluephagenesis* strains TD01 and TQ10 did not display significant growth under identical growth conditions as *H. rowanensis*, suggesting there was insufficient *Halomonas*-specific carbon sources available to support heterotrophic growth. Comparative heterotrophic growth of *H. rowanensis* was performed in wastewater supplemented with glycerol, which showed around 2.5-fold higher biomass production than (potential) chemoautotrophic growth (Additional file 1: Fig. S3).

**Fig. 3:**
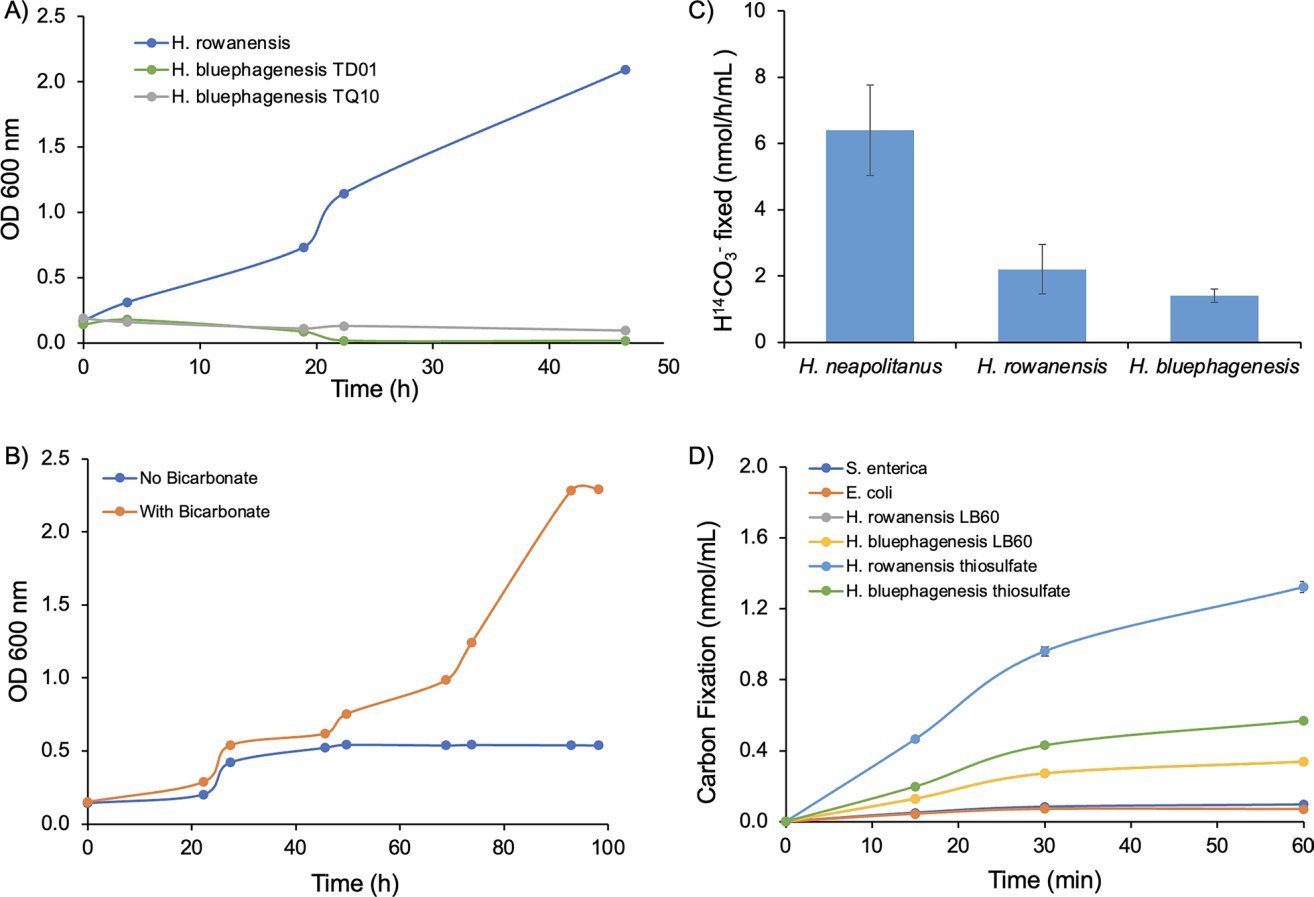
Chemoautotrophic growth of *H. rowanensis*. **A** Chemoautotrophic growth of three *Halomonas* species. Cultures were grown in thiosulfate growth medium containing 150 mM NaHCO_3_ at 30 °C for 48 h. **B** Growth of *H. rowanensis* with or without supplemental inorganic carbon. Cultures were grown in thiosulfate medium in the presence and absence of 150 mM NaHCO_3_ for 100 h at 30 °C. **C** Radiolabelled carbon fixation of non-permeabilised cultures. Cultures were grown in species-specific growth medium and the ^14^C (H^14^CO_3_^−^) fixation assay was performed as described previously [37] with the culture at OD 600 nm = 4.0. **D** Time course of ^14^C labelled bicarbonate fixation in six microorganisms. Cells (OD 600 nm = 4.0) were permeabilised with alkyltrimethylammonium bromide with NaH^14^CO_3_ addition to allow the measurement of total enzymatic carbon fixation activity independently of bicarbonate uptake systems. The *H. bluephagenesis* strain for parts **C**, **D** was TD1.0

Thermotolerant *Halomonas stevensii* is known to fix CO_2_ using thiosulfate as a sole energy source [21]. We further investigated both *H. rowanensis* and the *H. bluephagenesis* strains TD1.0 and TQ10 to determine if they are capable of chemoautotrophic or mixotrophic growth phenotypes using thiosulfate as the sole energy source [21]. After repeated subculturing into organic carbon-free thiosulfate medium, only *H. rowanensis* was capable of significant growth (Fig. 3a). The addition of NaHCO_3_ to the medium resulted in an enhancement in overall biomass production (Fig. 3b), suggesting *H. rowanensis* can fix CO_2_ into organic carbon [37, 43], similar to the growth of *H. stevensii* under chemoautotrophic conditions [21]. Given that *H. rowanensis* also grows efficiently under heterotrophic conditions, this suggests this organism may be a facultative chemoautotroph.

To assess the potential of CO_2_ fixation by *H. rowanensis*, we performed radiolabelling experiments with ^1^H^14^CO_3_^−^ and looked for label incorporation into cellular biomass. The NaH^14^CO_3_ was fed externally during growth, so label incorporation is a measure of both the efficiency of bicarbonate uptake systems as well as carbon fixation and retention intracellularly. Radiolabelled bicarbonate incorporation was detected for *H. rowanensis* (2.21 ± 0.75 nmol/h/mL at OD 600 nm = 4.0; Fig. 3c), which is equivalent to 2.47 ± 0.84 nmol/h/g cells. This is approximately threefold lower than label incorporation under the same growth conditions by a known chemoautotroph *H. neapolitanus* (6.40 ± 1.36 nmol/h/mL). Interestingly, *H. bluephagenesis* TD1.0 also displayed ^14^C incorporation (1.42 ± 0.20 nmol/h/mL), despite a lack of growth in thiosulfate medium. This suggests it contains the machinery for carbon fixation, but not via thiosulfate as an energy source.

Further radiolabelled bicarbonate incorporation studies were performed with *H. rowanensis* and *H. bluephagenesis* TD1.0 under heterotrophic (LB60) and chemoautotrophic (thiosulfate) growth conditions at pH 7.0. In this case, cells were permeabilised to remove the dependence on bicarbonate uptake into the cells. The highest levels of ^14^C incorporation were seen with *H. rowanensis* cultivated under chemoautotrophic conditions (1.32 ± 0.03 nmol/mL), with a near fourfold reduction when grown initially in heterotrophic growth medium (0.34 ± 0.01 nmol/mL; Fig. 3d). This suggests a full carbon fixation pathway may be upregulated when the culture is transitioned into autotrophic growth medium, rather than the organism simply containing a few genes enabling it to survive brief periods of fixed carbon starvation.

Radiolabelled bicarbonate incorporation was apparent with *H. bluephagenesis* TD1.0 during the 1 h assay despite its lack of growth in thiosulfate-base chemoautotrophic medium. The incorporation was less efficient that *H. rowanensis* (0.57 ± 0.01 nmol/mL; Fig. 3d), but also showed the upregulation when cells were transferred into chemoautotrophic medium. This suggests that carbon fixation may be a common trait within the *Halomonas* genus.

### Putative carbon fixation pathway

We examined the annotated genome sequences to detect potential pathways supporting both heterotrophic and carbon fixation metabolism. This was performed using an updated annotation of the genome with accuracies improved using BLAST sequence homology paired with RAST annotation. In addition, the overall enzyme class was confirmation in addition to the likely presence of the appropriate active site residues/arrangement via AlphaFold structural modelling [32]. As expected, the annotated genome contained all the expected genes required to support a heterotrophic lifestyle. This included complete pathways for glycolysis, TCA cycle with glyoxylate shunt, pentose phosphate and Entner–Duodoroff pathways [44]. Genes for a fully functional aerobic electron transport chain were also present, such as cytochrome c oxidase for utilising oxygen as a terminal electron acceptor and a proton translocating ATP synthase for chemiosmotic energy generation. The potential use of nitrate as an alternative terminal electron acceptor was also inferred by genes for respiratory nitrate reductase as well as genes permitting electron transfer from donor compounds formate and glycerol-3-phosphate.

Classic chemoautotrophic bacteria, such as *H. neapolitanus*, fix CO_2_ via the Calvin–Bensen–Bassham cycle, with energy supplied by sulfur oxidation pathways [45]. A carbon-concentration mechanism is present in *H. neapolitanus* via the presence of bicarbonate transporters, carbonic anhydrase and α-carboxysomes, which enclose the key carbon fixation enzyme ribulose 1,5-bisphosphate carboxylase/oxygenase (RubisCO) [46]. However, annotation of the *H. rowanensis* genome did not reveal the presence of any gene involved in the Calvin–Bensen–Bassham cycle or evidence of α-carboxysome formation. The quinoprotein dehydrogenase-associated SoxYZ-like carrier gene was found in the genome of *H. bluephagenesis* TD1.0, but no further genes involved in α-carboxysome formation were annotated.

**Fig. 4:**
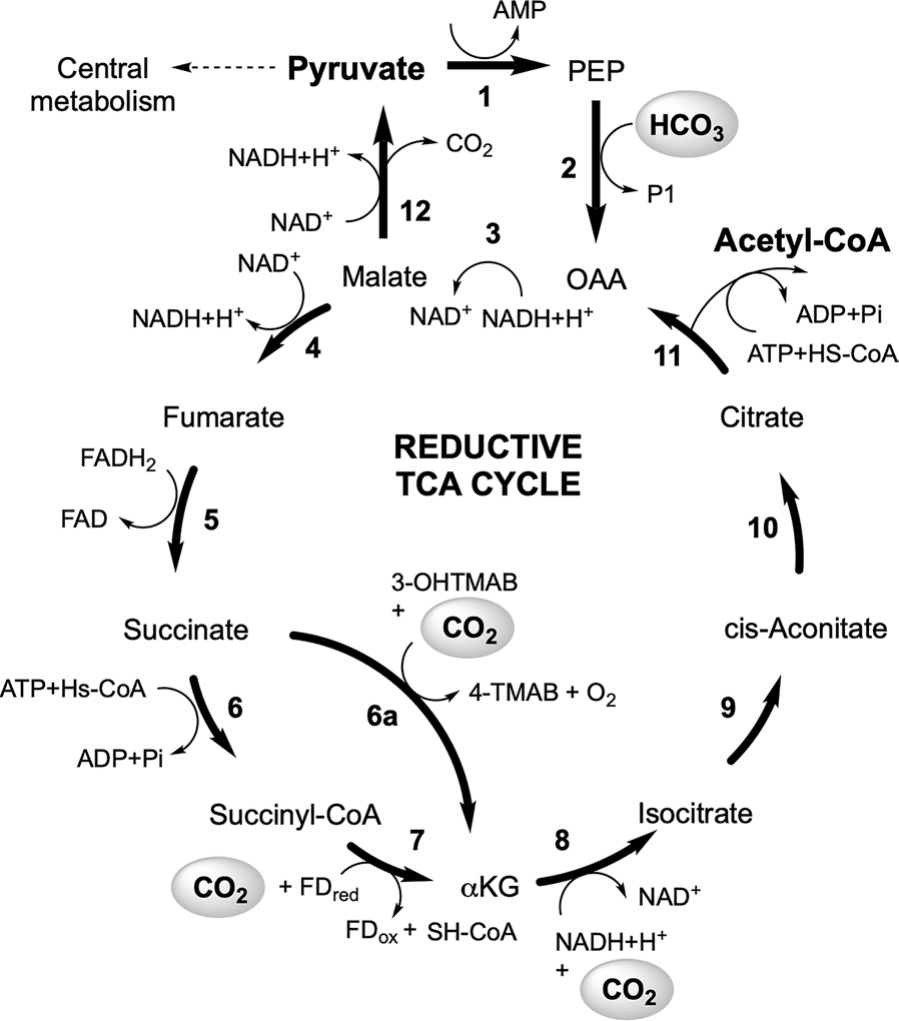
Putative carbon fixation pathway in *H. rowanensis*. Enzymes: 1 = phosphoenolpyruvate synthase; 2 = phosphoenolpyruvate carboxylase; 3 = malate dehydrogenase; 4 = fumarate hydratase; 5 = succinate dehydrogenase; 6 = succinyl-CoA ligase; 6a = γ-butyrobetaine,2-oxoglutarate dioxygenase; 7 = 2-oxoglutarate synthase; 8 = Isocitrate dehydrogenase [NADP]; 9–10 = aconitate hydratase; 11 = citrate lyase and 12 = NADP-dependent malic enzyme. Metabolites: PEP = phosphoenolpyruvate; αKG = α-ketoglutarate; 3-OHTMAB = 3-hydroxy-4-trimethylammoniobutanoate and 4-TMAB = 4-trimethylammoniobutanoate. Gene annotation was performed using a combined approach with Rapid Annotations using Subsystems Technology toolkit (RASTtk) along with BLASTp validation, using the KEGG carbon fixation pathways as guide for enzyme discovery. EC numbers for each enzyme can be found in Additional file 1: Table S3

The new annotated genome identified a putative complete reductive tricarboxylic acid (rTCA) cycle in *H. rowanensis* (Fig. 4) [47], which could account for the ability to fix CO_2_. This pathway has been found in other chemoautotrophs, such as *Chlorobium*, *Desulfobacter hydrogenophilus* and some members of the thermophilic Aquificales order and archaeal Thermoproteaceae family [48]. The rTCA cycle generates one molecule of oxaloacetate from four molecules of CO_2_ and requires 4–5 mol of adenosine 5′-triphosphate (ATP) [48]. Essential genes for this pathway are required to overcome key energetically unfavourable reverse reaction steps. This includes ATP citrate lyase, which catalyzes the cleavage of citrate into acetyl-CoA and oxaloacetate in a CoA- and ATP-dependent manner (Step 11 of Fig. 4). Other key enzymes are two of the four carbon dioxide-fixing enzymes 2-oxoglutarate:ferredoxin oxidoreductase (Step 7 of Fig. 4), and pyruvate:ferredoxin oxidoreductase (Additional file 1: Table S3) [48].

We identified at least one annotated gene for each step of the rTCA cycle (Additional file 1: Table S3), whose likely function were predicted by AlphaFold structural simulation and the DALI structural homology webserver (Additional file 1: Fig. S4). Other genes identified included enzymes catalysing the interconversion of malate to glyoxylate and acetyl CoA or pyruvate and those involved in the hydroxybutyrate [49] and hydroxypropionate [50] cycles. We also identified a gene for γ-butyrobetaine hydroxylase, which could potentially act as an alternative enzyme to catalyse steps 6–7 (succinate to α-ketoglutarate) in the presence of ascorbate [51]. Given the presence of key enzymes citrate lyase, NADPH dependent malic enzyme and malate dehydrogenase, the interconversion of citrate and pyruvate is likely possible.

### Putative thiosulphate utilisation pathways in *H. rowanensis*

Chemolithoautrophic bacterium obtain energy to fix atmospheric CO_2_ typically by extracting electrons from reduced ferrous (Fe[II]), thiosulfate (S_2_O_3_^−2^) or ammonium (NH_4_^+^) ions [52]. For example, energy generation in *H. neapolitanus* and *Paracoccus pantrophus* proceeds via the oxidation of thiosulfate to sulfate using the Sox system [53, 54], delivering eight electrons to the quinone pool for ATP generation (Additional file 1: Fig. S5a). Alternatively, some chemo-organoheterotrophic bacteria, including *Halomonas* species, are known to derive energy from the oxidation of thiosulfate to tetrathionate (Additional file 1: Fig. S5b) via the action of thiosulfate dehydrogenase (tsaD) [55].

Annotation of the genomes of *H. rowanensis* and *Halomonas* strain I4 revealed the presence of a variety of genes involved in the transport of thiosulfate to sulfite, sulfide and elemental sulfur (Additional file 1: Table S4). However, there were no Sox genes system or *tsaD* annotated in either genome that could explain how *H. rowanensis* could derive energy to fix CO_2_ in chemoautotrophic thiosulfate medium. Given the relative incompleteness of the *H. rowanensis* genome compared to *H. bluephagenesis* TD01, it is likely that the genes involved in chemoautotrophic energy generation are buried within the putative proteins. Therefore, further work is required before the mechanism(s) of energy generation is determined for *H. rowanensis* and *H. bluephagenesis* TD01.

**Fig. 5:**
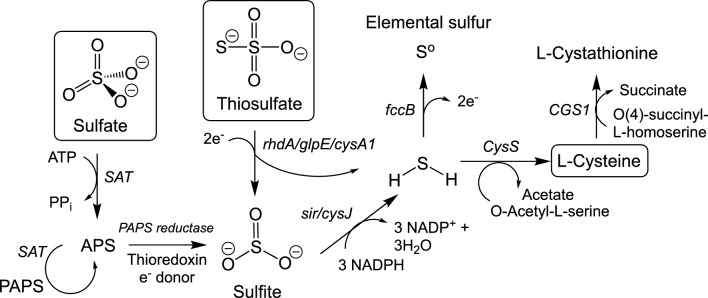
Putative thiosulfate and other oxidised sulfur utilisation pathways in *H. rowanensis*. APS = adenosine-5′-phosphosulfate; PAPS = 3'-phosphoadenylyl sulfate; Enzymes: *CGS1* = cystathionine γ-synthase; *CysS* = cysteine synthase; fccB = flavocytochrome c; rhdA/glpE/cysA1 = thiosulfate sulfur transferases; *SAT* = sulfate adenylyltransferase; *sir/cysJ* = sulfite reductase

The presence of multiple thiosulfate transferases and sulfate adenyltransferases suggest *H. rowanensis* contains the assimilatory and dissimilatory sulfate reduction and oxidation pathways. The sulfur metabolism genes annotated in *H. rowanensis* suggests they play a role as a sulphur source for l-cysteine and related compounds (Fig. 5). For example, several putative thiosulfate sulfur transferases (e.g., rhdA and glpE) could reduce thiosulfate to sulfite [56]. Further NADPH-dependent reductions could lead to the production of sulfides, which can act as a sulfur donor to O-acetyl-l-serine to generate l-cysteine [57]. Genes for the conversion of sulfate to sulfite via adenosine-5′-phosphosulfate (APS) are also present (Fig. 5). As these pathways overall are net energy requiring, they are unlikely to be coupled directly to CO_2_ fixation.

### Chemoautotrophic propane, ectoine and PHA production

We wished to test the potential of chemoautotrophic *H. rowanensis* to act as a robust microbial chassis to produce secondary products by incorporating an established route towards bio-propane (bio-LPG) production. This was chosen as propane production in *Halomonas* is already established in the literature [2, 20, 36], so could be used as a basis of comparison for the new strain. This was performed by incorporating the recombinant fatty acid photodecarboxylase variant G462V from *Chlorella variabilis* NC64A (CvFAP_G462V_). This enzyme catalyses the decarboxylation of butyric acid to propane [58], and was previously shown to be functionally expressed in *H. bluephagenesis* TQ10 cultivated on biodiesel waste glycerol and amino acids [2, 17, 20]. A second strain *H. bluephagenesis* TD1.0 also generated bio-propane with a higher degree of fitness [36]. However, both strains required extensive pH control measures to prevent the culture from its natural tendency to shift the pH up to 9.0, which would rapidly abolish propane production [2]. By incorporating CvFAP_G462V_ into *H. rowanensis*, we wished to investigate whether this neutral pH chassis would be more suitable for bio-propane production. We expressed CvFAP_G462V_ in *H. rowanensis* under control of a weak (P7) or strong (P102) constitutive promoter and cultivated the organism under blue light to activate CvFAP_G462V_ for propane production.

**Fig. 6:**
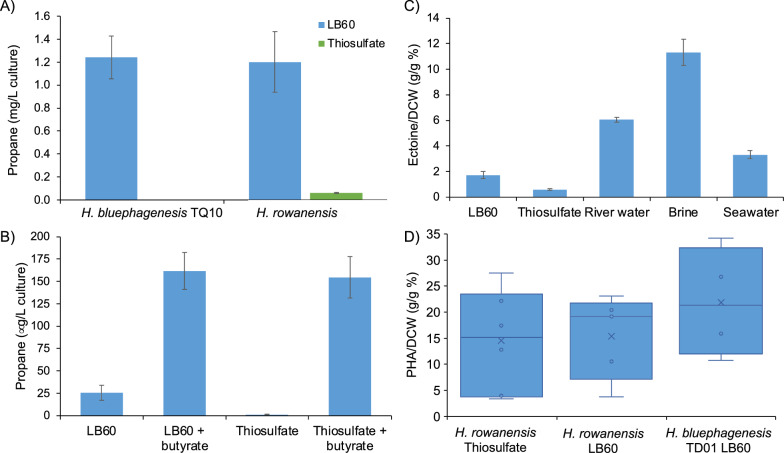
Propane production of *H. bluephagenesis* TQ10 and/or *H. rowanensis* expressing plasmid borne CvFAP_G462V_ with a (**A**) weak p7 and (**B**) strong p102 constitutive promoter. In part A, cultures (50 mL) were cultivated in LB60 medium (*H. bluephagenesis* TD1.0) or thiosulfate medium (*H. rowanensis*) for 6 h from a 1% inoculum at 30 °C and 200 rpm. Triplicate aliquots (1 mL) were sealed in 4 mL glass vials and incubated overnight at 30 °C and 180 rpm under a blue LED panel. For part **B**, *H. rowanensis* was cultivated as above in the two medium types with or without supplemental butyric acid (10 mM). Propane was quantified by Micro GC analysis of manual headspace samples. **C** Ectoine production of *H. rowanensis* in different growth media all with 10% (w/v) NaCl except the brine water which is ~ 20% NaCl. Cells were harvested by centrifugation and milked to release ectoine after 48 h in shake flasks at 30 °C. Ectoine and hydroxyectoine were quantified by HPLC. **D** Box plot of PHA production. PHB was accumulated over 48 h in LB60 and thiosulfate media in shake flasks at 30 °C. Cells from 50 mL were harvested by centrifugation and freeze dried. CDW was recorded and PHB concentration was determined by GC after derivatisation. Data are shown as the g PHB per g of freeze dried CDW starting material. HR = *H. rowanensis*; TD01 = *H. bluephagenesis* TD01 (wild-type isolate). The PHB titre and CDW data are available in Additional file 1: Table S2

Propane production from CO_2_ was more significant when using the weaker P7 promoter (61.8 µg/L culture; Fig. 6a). Supplementation with butyric acid (substrate and carbon source) led to a 19-fold increase in propane titres (1.2 mg/L culture). These relatively low titres compared to prior studies with heterotrophic *H. bluephagenesis* TQ10 reflects differences in the growth rates between heterotrophic and autotrophic conditions, likely differences in the intracellular butyrate concentration and relatively high apparent kinetic constant of CvFAP [2].

Surprisingly, switching to a higher strength promoter system yielded lower overall yields of propane under heterotrophic and chemoautotrophic growth conditions (Fig. 6b). This suggests the high expression of CvFAP_G462V_ may impart some toxicity on the system. Overall, propane titres of *H. rowanensis* from CO_2_ (thiosulphate medium without butyric acid) were around tenfold lower than photoautotrophic propane production by *Synechcocystis* PCC 6803 (~ 11.1 ± 2.4 mg propane/L/day) [2]. However, the latter strain had been bioengineered to accumulate small chain fatty acids by knocking out the native fatty acyl ACP synthase gene (∆*aas*) and to co-express CvFAP_G462V_ with a butyryl-ACP thioesterase from *Bacteroides fragilis* (Tes4) [59].

After establishing chemoautrophic and heterotrophic bio-propane production in *H. rowanensis*, we determined the levels of naturally occurring ectoine and PHA to see the potential of multiproduct generation. The highest titres of ectoine when *H. rowanensis* was cultivated on mineral enriched brine water with 1% (v/v) glycerol as the carbon source (11.31 ± 1.03 ectoine/DCW (%); Fig. 6c). Other enriched medium based on natural water systems gave good titres of ectoine (Fig. 6c). Cells grown chemoautotrophically on CO_2_ produced 20-fold lower titres of ectoine, with the degree of salinity determined to be important for ectoine production (Fig. 2B and Additional file 1: Fig. S6). Ensuring sufficiently high salinity under chemoautotrophic conditions will likely improve ectoine titres in *H. rowanensis.*

PHA production by *H. rowanensis* was essentially the same when cultivated under heterotrophic (LB60) or chemoautotrophic (thiosulfate) conditions (15.38 ± 8.01 vs 14.54 ± 9.72% PHA/DCW; Fig. 6d). These titres are slightly lower than comparable heterotrophic growth of *H. bluephagenesis* TD1.0 (21.90 ± 10.56% PHA/DCW), in the absence of glucose feeding. Therefore, optimal titres of PHA are generated under heterotrophic condition, where excess organic carbon can be supplied to stimulate PHA accumulation.

Further studies are required to optimise the CvFAP construct for *H. rowanensis* to increase recombinant propane titres in addition to naturally producing ectoine and PHA. Supplementing cultures with butyric acid is likely to switch the metabolism towards heterotrophic growth as *Halomonas* species are known to grow on butyric acid as a carbon source [2]. Therefore, while chemoautotrophic propane, ectoine and PHA have been demonstrated, it is likely that switching to conventional glucose fed and butyric acid supplemented cultivation will increase the titres of all three target compounds.

## Conclusions and future perspectives

The recruitment of environmental isolates as de novo microbial chassis is not a new concept. The practice of screening for microorganisms of a specific genera or growth condition can sometimes lead to surprising growth phenotypes, which can open alternative approaches to achieve the desired bioproduction goal. The discovery of facultative chemoautotrophic *H. rowanensis* with relatively rapid growth on inorganic carbon sources and wastewater raises the possibility of dramatically reducing the carbon footprint of chemicals and fuels bioproduction by utilising industrial waste gases or bicarbonate from carbon capture technologies as the carbon (CO_2_) and energy (sulphur compounds) sources. This gives it an advantage as a potential robust industrial chassis for secondary product formation once it has been optimised to increase titres. The neutral pH optimum allows it to more easily express enzyme pathways that are not active at the high pH that *H. bluephagenesis* thrives at. The broader salt tolerance of *H. rowanensis* compared to *H. bluephagenesis* strains allows for non-sterile cultivation to be possible even at pH 7.0 as the culture thrives at the higher salinity of 10%.

Thiosulfate and inorganic sulphur contaminated wastewater can be found in the mining industry [60], steelworks [61], offshore gas, shale oil [62] and water dichlorination processes [63]. A potential scenario could be the capture of both industrial CO_2_ from the flue gas and thiosulfate-contaminated water found at steel mills as the basis for a bioproduction medium. This would effectively be a means to bioremediate thiosulfate while capturing CO_2_ emissions as both biomass and propane.

There are considerable cost savings to be achieved by exploiting the ability of halophiles to be cultivated under non-sterile growth conditions without the need for fresh water. The relative ease at which the *Halomonas* genetic toolbox was transmittable to an unmodified natural isolate highlights the potential of environmental screening to discover more unique potential microbial chassis. Overall, the utilisation of a chemoautotrophic halophilic industrial microbial chassis could improve the sustainability of the process in addition to an overall reduction in the carbon footprint. The generation of valuable biochemicals and fuels from CO_2_ could increase the energy security and could contribute towards global carbon management targets and help achieve clean air directives.

### Supplementary Information

**Additional file 1**: **Figure S1**. Plasmid map for propane production in *Halomonas*. **Figure S2**. Tolerance of *Halomonas* isolates for salinity, pH and butyric acid. **Figure S3**. Growth of *H. rowanensis* in mineral-based media using polluted water with and without exogenous carbon sources. **Figure S4**. Superimposition of AlphaFold predicted structures of reverse TCA cycle proteins and the closest DALI homology match. **Figure S5**. Sulfur oxidation systems for energy generation. **Figure S6.** Ectoine production by *Halomonas rowanensis* in the thiosulfate minimal medium with a range of salinity. **Table S1**. Partial 16S rDNA sequence analysis of ‘Old Biot’ brine spring isolates. **Table S2**. Extended PHB assay data for Figs. 2C and . **Table S3**. Putative carbon fixation cycle genes identified within the genome of *H. rowanensis* by protein sequence homology and AlphaFold structural homology. **Table S4.** Putative sulfur metabolism genes identified within the genome of *H. rowanensis* by protein sequence homology and AlphaFold structural homology.

## Data Availability

The latest annotated *H. rowanensis* genome sequence is available on GenBank (Bioproject ID PRJNA994417) and ENA (PRJEB64241, ERP149380), project number and project title, accession number pending) as is *H. bluephagensis* TD01 (Genbank, GCA_923868895.1 and GCA_000219565.1). *Halomonas rowanensis* has been deposited with the UKHSA patent collection (https://www.culturecollections.org.uk/deposit-with-us/patent-deposits/patent-deposit-service.aspx) under Deposit Reference 23,021,001.

## Abbreviations

16S: 16 Svedberg (sedimentation rate)
CvFAP: Fatty acid photodecarboxylase from *Chlorella variabilis* NC64A
LCA: Life cycle analysis
LED: Light emitting diode
PHA: Polyhydroxyalkanoate
TCA cycle: Tricarboxylic acid cycle
TEA: Technoeconomic analysis
