# Supplementary material for: Chemoautotrophic production of gaseous hydrocarbons, bioplastics and osmolytes by a novel Halomonas species
Source: Biotechnol Biofuels Bioprod. 2023 Oct 11;16:152. doi: 10.1186/s13068-023-02404-1 (PMC10568851; doi:10.1186/s13068-023-02404-1)
Supplement: Supplementary file 1 — Additional file 1: Figure S1. Plasmid map for propane production in Halomonas. Figure S2. Tolerance of Halomonas isolates for salinity, pH and butyric acid. Figure S3. Growth of H. rowanensis in mineral-based media using polluted water with and without exogenous carbon sources. Figure S4. Superimposition of AlphaFold predicted structures of reverse TCA cycle proteins and the closest DALI homology match. Figure S5. Sulfur oxidation systems for energy generation. Figure S6. Ectoine production by Halomonas rowanensis in the thiosulfate minimal medium with a range of salinity. Table S1. Partial 16S rDNA sequence analysis of ‘Old Biot’ brine spring isolates. Table S2. Extended PHB assay data for Figs. 2C and . Table S3. Putative carbon fixation cycle genes identified within the genome of H. rowanensis by protein sequence homology and AlphaFold structural homology. Table S4. Putative sulfur metabolism genes identified within the genome of H. rowanensis by protein sequence homology and AlphaFold structural homology. [file 13068_2023_2404_MOESM1_ESM.docx]

**Additional file 1**

**Chemoautotrophic Production of Gaseous Hydrocarbons, Bioplastics and Osmolytes by a Novel *Halomonas* Species**

Matthew Faulkner^a,^, Robin Hoeven^c,d^, Paul P. Kelly^a,^, Yaqi Sun^b^, Helen Park^a^, Lu-Ning Liu^b^, Helen S. Toogood ^a,c,*^ & Nigel Scrutton^a,c,*^

^a^Manchester Institute of Biotechnology, The University of Manchester, 131 Princess Street, Manchester M1 7DN, United Kingdom

^b^Institute of Systems, Molecular and Integrative Biology, University of Liverpool, Biosciences building, Crown Street, Liverpool L69 7BE, UK.

^c^C3 Biotechnologies Ltd, 20 Mannin Way, Caton Road, Lancaster, Lancashire LA1 35W, UK

^d^Present address: Engineering Building A, University of Manchester, Oxford Road, Manchester M13 9PL, United Kingdom.

**Table of Contents**

| **FIGURES** |  |  |
| --- | --- | --- |
| **Figure S1** | Plasmid map for propane production in *Halomonas* | 2 |
| **Figure S2** | Tolerance of *Halomonas* isolates for salinity, pH and butyric acid | 3 |
| **Figure S3** | Growth of *H. rowanensis* in mineral-based media using polluted water with and without exogenous carbon sources | 4 |
| **Figure S4** | Superimposition of AlphaFold predicted structures of reverse TCA cycle proteins and the closest DALI homology match | 5 |
| **Figure S5** | Sulfur oxidation systems for energy generation | 6 |
| **Figure S6** | Ectoine production by *Halomonas Rowanensis* in the thiosulfate minimal medium with a range of salinity | 6 |
|  |  |  |
| **TABLES** |  |  |
| **Table S1** | Partial 16S rDNA sequence analysis of ‘Old Biot’ brine spring isolates | 7 |
| **Table S2** | Extended PHB assay data for Figures 2C and Figure 6D | 7 |
| **Table S3** | Putative carbon fixation cycle genes identified within the genome of *H. rowanensis* by protein sequence homology and AlphaFold structural homology | 8 |
| **Table S4** | Putative sulfur metabolism genes identified within the genome of *H. rowanensis* by protein sequence homology and AlphaFold structural homology | 9 |
| **References** | | 10 |

**Additional file 1: Figures**


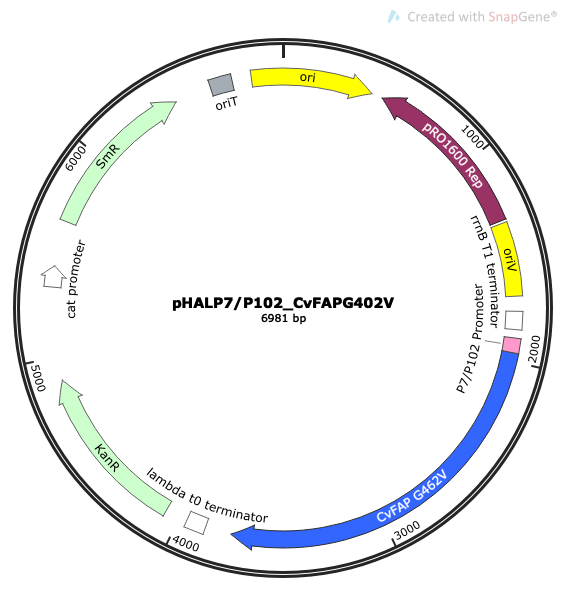


**Figure S1.** Plasmid map for propane production in *Halomonas*. The CvFAP_G402V_ promoter region (pink) differs between the two plasmids by the sequence of the constitutive promoter (P7 or P102) [1].


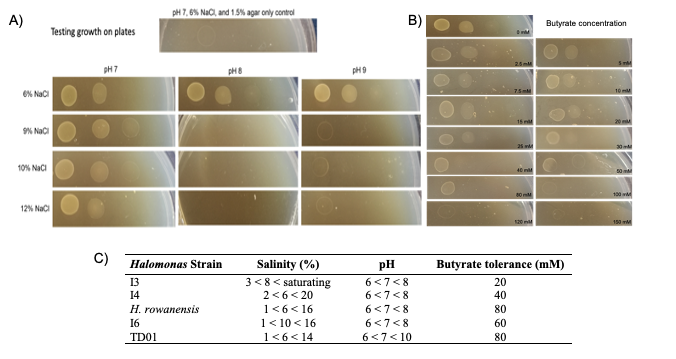


**Figure S2.** Tolerance of *Halomonas* isolates for salinity, pH and butyric acid. Isolates were cultivated on LB-based medium with variations in the A) pH (7-9), NaCl concentration (6-12%) and B) butyric acid (0-80 mM). Cultures were grown at 37 °C for 16 h. The growth images in A) and B) the growth screen of *Halomonas rowanensis* varying pH, salinity and butyrate concentration C) Data is expressed as the minimum, optimal and maximum conditions of pH/salinity where visible growth was observed. The butyrate concentrations indicate the maximum levels tolerated before growth was retarded. *H. rowanensis* is also known as *Halomonas* isolate I5. TD01 refers to *Halomonas bluephagenesis* TD01, the wild-type isolate [2] that was the precursor to the TD1.0 and TQ10 strains. These data were collected on all wild-type strains that have not undergone any genetic manipulations.


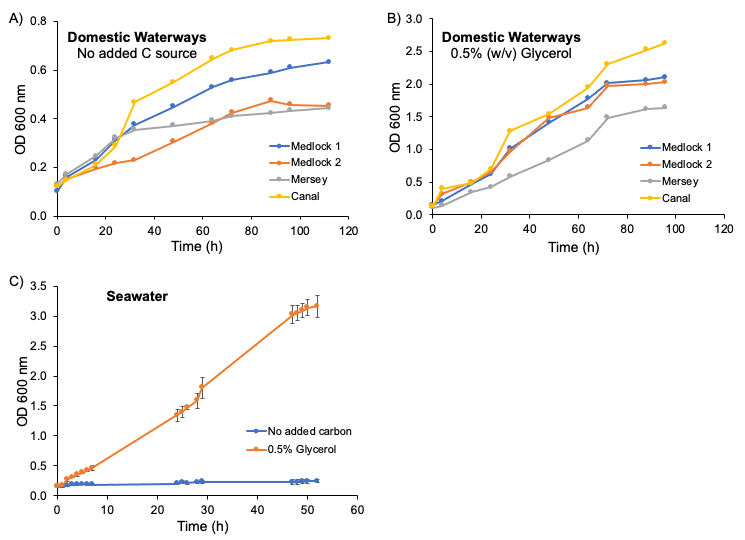


**Figure S3.** Growth of *H. rowanensis* in mineral-based media using polluted water with and without exogenous carbon sources. Growth of *H. rowanensis* in environmental water samples from the Greater Manchester region (UK) in the A) absence or B) presence of a supplemental carbon source. C) Growth of *H. rowanensis* in sea water (Irish sea) with or without supplemental carbon source (0.5% glycerol). Cultures were grown for up to 120 h at 30 °C in sanitised and filtered polluted water containing additional minerals/NaCl required for halophilic growth with or without 0.5% (w/v) glycerol. An average of 3 independent biological repeats of were performed in part C) with error bars representing 1 standard deviation of the data. The Greater Manchester water samples were obtained from the Medlock and Mersey rivers and the Rochdale canal (UK).


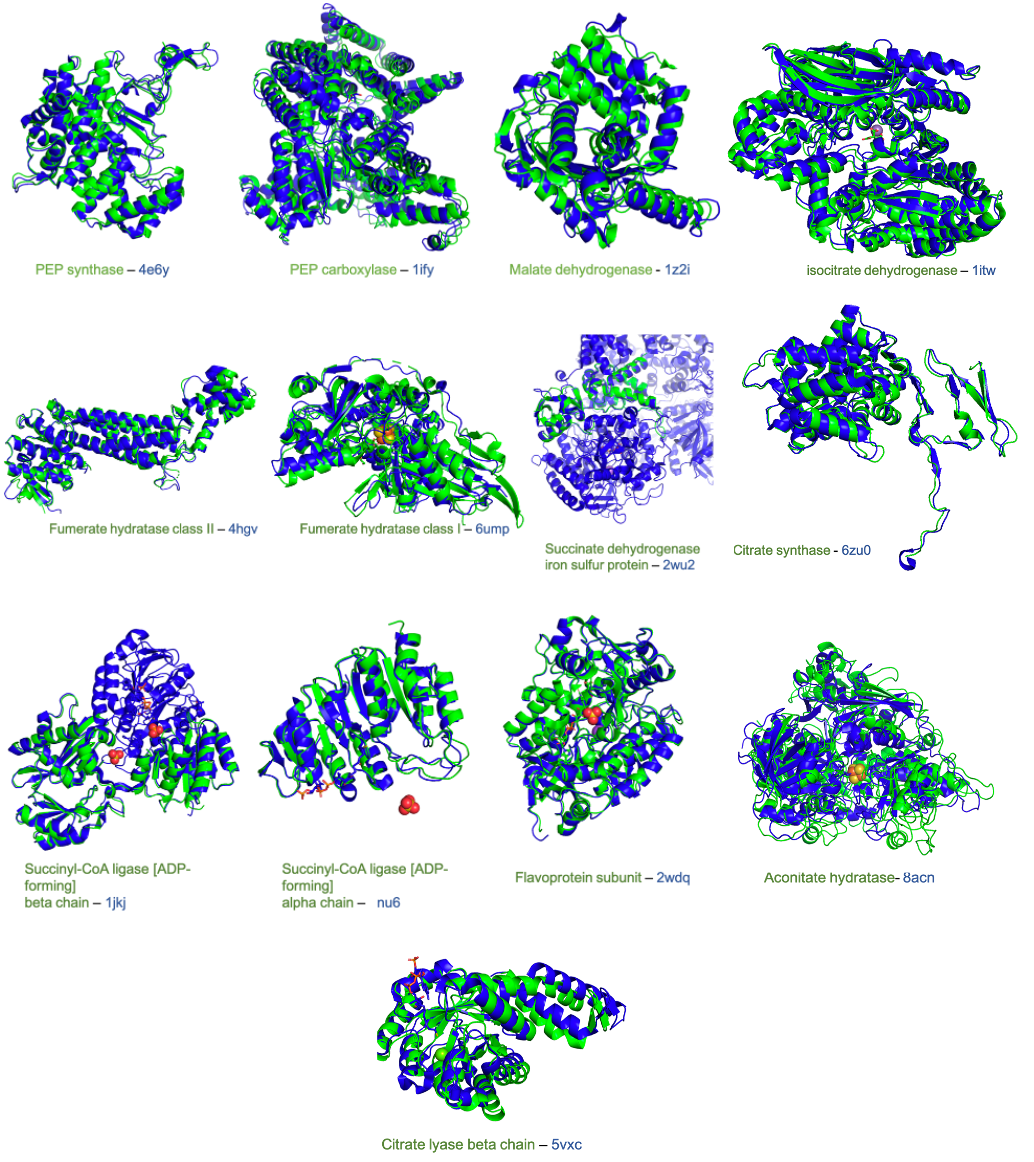


**Figure S4.** Superimposition of AlphaFold predicted structures of reverse TCA cycle proteins (green) and the closest DALI homology match (blue). The PDB code for the homology model is shown in blue and the inferred gene name for the *Halomonas* protein sequence is shown in green. The AlphaFold structure prediction did not include the presence of potential co-factors. Structures were aligned using Pymol [3].


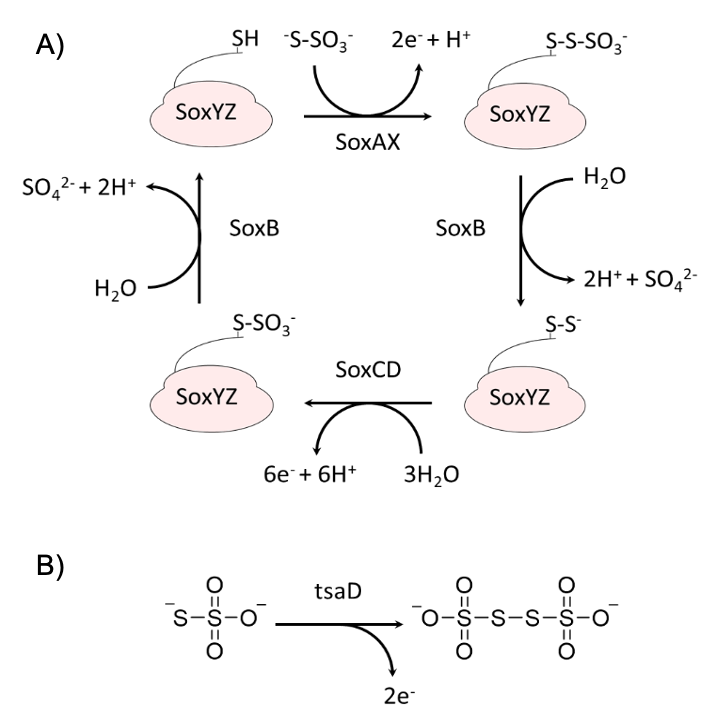


**Figure S5**: Sulfur oxidation systems for energy generation. A) Sox system for thiosulfate oxidation to sulphate in *Halothiobacillus*. Eight electrons per thiosulfate enter the quinone pool. B) Thiosulfate dehydrogenase (tsaD) c-type cytochrome for oxidation of thiosulfate to tetrathionate.

**Figure S6:** Ectoine production by *H. rowanensis* in thiosulfate minimal medium with a range of salinity from 2-15% (w/v) NaCl. Cultures were grown at 30 °C for 48 h in the corresponding medium.

**Additional file 1: Tables**

| **Table S1** Partial 16S rDNA sequence analysis of ‘Old Biot’ brine spring isolates. | | |
| --- | --- | --- |
| **Isolate IDs** | **Nearest relative* (% Identity)** | **Natural source (ref.)** |
| I1 | *Bacillus hwajinpoensis* strain SW-72 (100%) | Sea water, Korea ^21^ |
| I3, I4, I5, I6 and I7 | *Halomonas taeanensis* strain BH539 (98%) | Solar saltern, Korea ^22^ |
| I8 | *Rothia kristinae* (aka *Kocuria kristinae*) strain DSM 20032 (100%) | Skin ^23^ |
| I9 and I10 | *Idiomarina loihiensis* strain L2TR (99%) | Deep sea hydrothermal vent, Hawaii ^24^ |
| *Partial 16S rDNA sequences were submitted to a BLASTn search against database ‘16S ribosomal RNA sequences (Bacteria and Archaea)’(<https://www.ncbi.nlm.nih.gov/>). | | |

| **Table S2**. Extended PHB assay data for Figures 2C and 6D. | | | |
| --- | --- | --- | --- |
| Sample (strain / medium / replicate) | PHB (%) | PHB (mg/L) | CDW (mg/L) |
| HR thiosulfate 1 | 3.38 | 8.12 | 240 |
| HR thiosulfate 2 | 3.97 | 25.43 | 640 |
| HR thiosulfate 3 | 22.13 | 70.83 | 320 |
| HR thiosulfate 4 | 27.47 | 87.90 | 320 |
| HR thiosulfate 5 | 12.84 | 35.95 | 280 |
| HR thiosulfate 6 | 17.44 | 69.74 | 400 |
| HR LB60 1 | 10.55 | 116.08 | 1100 |
| HR LB60 2 | 20.39 | 187.61 | 920 |
| HR LB60 3 | 3.76 | 36.11 | 960 |
| HR LB60 4 | 23.05 | 138.32 | 600 |
| HR LB60 5 | 19.14 | 126.30 | 660 |
| TD01 LB60 1 | 10.74 | 126.77 | 1180 |
| TD01 LB60 2 | 15.90 | 193.97 | 1220 |
| TD01 LB60 3 | 26.79 | 417.88 | 1560 |
| TD01 LB60 4 | 34.15 | 239.07 | 700 |
| TD01 LB60 5 | 27.81 | 411.72 | 1480 |
| HR 4% glucose 1 | 37.87 | 5280.76 | 2000 |
| HR 4% glucose 2 | 67.36 | 1212.46 | 1800 |
| HR 4% glucose 3 | 36.36 | 4399.99 | 1600 |
| HR 4% glucose 4 | 50.59 | 1113.03 | 2200 |
| TD01 4% glucose 1 | 81.34 | 4179.94 | 3400 |
| TD01 4% glucose 2 | 48.01 | 1728.35 | 3600 |
| TD01 4% glucose 3 | 66.99 | 4478.31 | 3000 |
| TD01 4% glucose 4 | 69.69 | 2648.33 | 3800 |
| HR = *H. rowanensis*; TD01 = *H. bluephagenesis* TD01. | | | |

| **Table S3.** Putative carbon fixation cycle genes identified within the genome of *H. rowanensis* by protein sequence homology and AlphaFold structural homology. | | | |
| --- | --- | --- | --- |
| **Step(s)** | **EC number** | **Annotation** | **Enzyme name** |
| 1 | 2.7.9.2 | CDS.2243 | Phosphoenolpyruvate synthase^a^ |
| 2 | 4.1.1.31 | CDS.904 | Phosphoenolpyruvate carboxylase^b^ |
| 3 | 1.1.1.37 | CDS.419 | Malate dehydrogenase [NAD]^c^ |
| 4 | 4.2.1.2 | CDS.2291 | Fumarate hydratase class II^d^ |
| 5 | 1.3.5.1 | CDS.140 | Succinate dehydrogenase iron-sulfur protein [quinone/related acceptor]^e^ |
| 6 | 6.2.1.5 | CDS.2447, CDS.2448 | Succinyl-CoA ligase [ADP-forming] beta chain,  Succinyl-CoA ligase [ADP-forming] alpha chain |
| 6a | 1.14.11.1 | CDS1671 | γ-Butyrobetaine hydroxylase: alternative steps 6-7 |
| 7 | 1.2.7.3 | *Not found* | 2-Oxoglutarate synthase |
| 8 | 1.1.1.42 | CDS.2542 | Isocitrate dehydrogenase [NADP] |
| 9-10 | 4.2.1.3 | CDS.1587 | Aconitate hydratase |
| 11 | 4.1.3.6 | CDS.1750 | Citrate lyase beta chain^f^ |
| 12 | 1.1.1.83 | CDS.344 | D-Malate dehydrogenase (decarboxylating), which could potentially catalyse this reaction^g^ |
| Annotated sequences were corroborated by the AlphaFold 2.0 [4] and the DALI [5] webservers.  ^a^CDS.1432 pyruvate kinase can also catalyse the same reaction.  ^b^CDS.172 is also annotated to be phosphoenolpyruvate carboxykinase [ATP].  ^c^CDS.2526 is annotated to be fumarate hydratase and could putatively catalyse this reaction.  ^d^CDS.2443/CDS.2442 were annotated to be iron sulfur proteins that could be involved in this reaction.  ^e^CDS.2441 is also annotated as potentially catalysing this reaction.  ^f^CDS.516/CDS.167 are annotated to be malate:quinone oxidoreductases, which catalyse the same reaction.  ^g^CDS.1629 was originally annotated as an NADP-dependent malic enzyme [NADP] (EC 1.1.1.40), but AlphaFold predicts it is a TRK system potassium uptake protein instead. | | | |

| **Table S4.** Putative sulfur metabolism genes identified within the genome of *H. rowanensis* by protein sequence homology and AlphaFold structural homology. | | | |
| --- | --- | --- | --- |
| **Abbrev.** | **EC number** | **Annotation** | **Enzyme name** |
| GlpE | 2.8.1.1 | CDS.697 | Thiosulfate sulfur transferase |
| GlpE | 2.8.1.1 | CDS.1427 | Thiosulfate sulfur transferase |
| - | 2.8.1.1 | CDS.148 | Thiosulfate sulfur transferase |
| - | 2.8.1.1 | CDS.1926 | Thiosulfate sulfur transferase |
| tusA | - | CDS.798 | Sulfur carrier protein |
| thiS | - | CDS.1385 | Sulfur carrier protein |
| fdhD | - | CDS.2842 | Sulfur carrier protein |
| - |  | CDS.242 | Sulfate transporter |
| - |  | CDS.2682 | Sulfate transporter |
| CysZ |  | CDS.1533 | Sulfate transporter |
| cysT |  | CDS.371 | Sulfate transport system permease |
| ssuC |  | CDS.2525 | Sulfonate transport permease |
| CysN  CysC | 2.7.7.5  2.7.7.5 | CDS.184  CDS.185 | Sulfate adenylyltransferase subunit 1  Sulfate adenylyltransferase subunit 2 |
| DsrE |  | CDS.2926 | Sulfurtransferase – dissimilatory sulfur oxidation pathway |
| tusE | 2.8.1 | CDS.2829 | Sulfur transferase |
| SIR | 1.8.7.1 | CDS.2859 | Sulfite reductase [ferredoxin] |
| MET3 | 2.7.7.4 | CDS.782 | ATP-sulfurylase/sulfate adenylate transferase |
| cysH | 1.8.4.8 | CDS.9 | Phosphoadenylyl-sulfate (PAPS) reductase [thioredoxin] |
| MET5 | 1.8.1.2 | CDS.3415 | Assimilatory sulfite reductase (NADPH) |
| CysS | 2.5.1.47 | CDS.1560 | Cysteine synthase |
| CGS1 | 2.5.1.48 | CDS.2005 | Cystathionine γ-synthase |
| fccB | 1.8.2.3 | CDS.1130 | Flavocytochrome c |
| pspE | 2.8.1.1 | CDS.765 | Thiosulfate sulfurtransferase |
| sseB | 2.8.1.1 | CDS.1126 | Thiosulfate sulfurtransferase |
| csdA | 2.8.1.7 | CDS.129 | Cysteine desulfurase |
| IscS | 2.8.1.7 | CDS.1606 | Cysteine desulfurase - tRNA sulfurtransferase complex |
| IscA | - | CDS.1607 | Iron binding [FeS] tRNA sulfurtransferase complex |
| Annotated sequences were corroborated by the AlphaFold 2.0 [4] and the DALI [5] webservers. | | | |

**References**

1. Trisrivirat D, Hughes JMX, Hoeven R, Faulkner M, Toogood H, Chaiyen P *et al.* Promoter engineering for microbial bio-alkane gas production. Synth Biol. 2020;5:ysaa022.

2. Chen X, Yin J, Ye J, Zhang H, Che X, Ma Y *et al.* Engineering *Halomonas bluephagenesis* TD01 for non-sterile production of poly(3-hydroxybutyrate-co-4-hydroxybutyrate). Bioresour Technol. 2017;244:534-41.

3. DeLano WL. The PyMOL User's Manual Palo Alto, CA, USA.: DeLano Scientific; 2002.

4. Jumper J, Evans R, Pritzel A, Green T, Figurnov M, Ronneberger O *et al.* Highly accurate protein structure prediction with AlphaFold. Nature. 2021;596:583-9.

5. Holm L. DALI and the persistence of protein shape Protein Sci. 2020;29:128-40.
